# Aberrant clones: Birth order generates life history diversity in Greater Duckweed, *Spirodela polyrhiza*

**DOI:** 10.1002/ece3.3822

**Published:** 2018-01-17

**Authors:** Hebah S. Mejbel, Andrew M. Simons

**Affiliations:** ^1^ Department of Biology Carleton University Ottawa ON Canada

**Keywords:** bet hedging, birth order, diversification, dormancy, phenotypic plasticity, *Spirodela polyrhiza*

## Abstract

Environmental unpredictability is known to result in the evolution of bet‐hedging traits. Variable dormancy enhances survival through harsh conditions, and is widely cited as a diversification bet‐hedging trait. The floating aquatic plant, *Spirodela polyrhiza* (Greater Duckweed), provides an opportunity to study diversification because although partially reliable seasonal cues exist, its growing season is subject to an unpredictable and literally “hard” termination when the surface water freezes, and overwinter survival depends on a switch from production of normal daughter fronds to production of dense, sinking “turions” prior to freeze‐over. The problem for *S. polyrhiza* is that diversified dormancy behavior must be generated among clonally produced, genetically identical offspring. Variation in phenology has been observed in the field, but its sources are unknown. Here, we investigate sources of phenological variation in turion production*,* and test the hypothesis that diversification in turion phenology is generated within genetic lineages through effects of parental birth order. As expected, phenotypic plasticity to temperature is expressed along a thermal gradient; more interestingly, parental birth order was found to have a significant and strong effect on turion phenology: Turions are produced earlier by late birth‐order parents. These results hold regardless of whether turion phenology is measured as first turion birth order, time to first turion, or turion frequency. This study addresses a question of current interest on potential mechanisms generating diversification, and suggests that consistent phenotypic differences across birth orders generate life history variation.

## 1 INTRODUCTION

An understanding of the mechanisms underlying organismal adaptation is required to explain species persistence and survival in variable environments. Organisms may respond directly to environmental change through (nonadaptive) physiological or behavioral plasticity (Cleland, Chuine, Menzel, Mooney, & Shwartz, 2007), or indirectly through various evolutionary mechanisms (Bell & Gonzalez, 2009; Charmantier & Garant, 2005; Simons, 2011). Evolutionary modes of response to environmental variance include adaptive tracking, adaptive phenotypic plasticity, and bet hedging (Philippi & Seger, 1989; Seger & Brockmann, 1987; Simons, 2011; Slatkin, 1974; Via & Lande, 1985). When a predictable relationship exists between trait expression and fitness across environments, organisms may express adaptive phenotypic plasticity, where adjustment of phenotypic expression has evolved to occur over short timescales (Bradshaw, 1965; Lloyd, 1984; Stearns, 1989, 1989; Via & Lande, 1985). The evolution of adaptive plasticity is limited by the availability of dependable environmental cues (van Kleunen & Fischer, 2005), by the availability of genetic variance for norms of reaction (Via & Lande, 1985), by physical, physiological and behavioral constraints, and also by possible costs associated with the expression of plasticity (Auld, Agrawal, & Relyea, 2009; van Kleunen & Fischer, 2005; Murren et al., 2015).

When environmental conditions are unpredictable, and under the broad array of circumstances under which plasticity is constrained, bet hedging is expected to evolve (Gillespie, 1974; Seger & Brockmann, 1987). Bet‐hedging traits evolve over the long term because they maximize geometric mean fitness despite reducing expected fitness over the shorter term (Dempster, 1955; Philippi & Seger, 1989; Seger & Brockmann, 1987). Bet hedging reduces intergenerational variance in fitness (Gillespie, 1974) and may occur either through diversification or through expression of conservative “safe” traits (Philippi & Seger, 1989; Seger & Brockmann, 1987; Simons, 2011). For example, organisms may diversify offspring phenotypes such as the timing of seed germination (Simons, 2009) if the fitness associated with timing cannot be predicted at the time the “decision” is made, ensuring that at least a fraction will succeed (Cohen, 1966; Seger & Brockmann, 1987). Conservative bet hedging is probably common but is more difficult to study (Simons, 2002); for example, timing of reproduction of semelparous (monocarpic) plants may be a conservative strategy but has the appearance of suboptimality in that it is restricted to a safe period early in the season, whereas reproduction at later dates would be optimal during “normal” seasons (Hughes & Simons, 2014; Simons & Johnston, 2003).

Because natural environmental variation has both predictable and unpredictable components, the joint evolution of phenotypic plasticity and bet hedging is expected (Donaldson‐Matasci, Bergstrom, & Lachmann, 2013; Gremer, Kimball, & Venable, 2016; Simons, 2014). Although our knowledge of the prevalence of bet‐hedging strategies is improving (Beaumont, Gallie, Kost, Ferguson, & Rainey, 2009; Graham, Smith, & Simons, 2014; Simons, 2011), our understanding of the mechanisms underlying the generation of diversification and of the joint expression of these two modes of response is underdeveloped.

Dormancy is commonly cited as an adaptation to environmental change as both a bet‐hedging strategy and as adaptive phenotypic plasticity (Gremer et al., 2016; Nilsson, Tuomi, & Astrom, 1996; Simons, 2014; ). Dormancy is a mechanism allowing escape from detrimental conditions and is expressed in a wide range of taxa, including microorganisms, plants, and animals (Belozerov, 2008; Shefferson, 2009; Sussman & Douthit, 1973). Seed dormancy is a mechanism in which viable seeds do not germinate—even under suitable environments—until dormancy requirements are satisfied (Baskin & Baskin, 1998; Shefferson, 2009). Although most commonly expressed in propagules of both animals (Furness, Lee, & Reznick, 2015) and plants (Clauss & Venable, 2000; Lu, Tan, Baskin, & Baskin, 2013), dormancy may occur at various life stages in different species. For example, vegetative dormancy occurs over a prolonged period where herbaceous perennial plants persist below ground, and delay sprouting for one or more seasons (Shefferson, 2009 **)**. Some organisms rely on environmental cues to trigger the onset and/or termination of dormancy. Cues that can influence dormancy behavior include photoperiod (Masuda, Urakawa, Ozaki, & Okubo, 2006), nutrient and moisture availability (Baskin & Baskin, 1998), and temperature (Heide, 2011), with response to particular cues such as photoperiod being species‐specific (Heide, 2011; Masuda et al., 2006).

Because a cue at the time a germination “decision” is made is only a partially reliable indication of conditions for seedling establishment, germination is an example of a trait that is expected to be regulated by both plasticity and diversification bet hedging (Clauss & Venable, 2000; Lloyd, 1984; Simons, 2014). In desert annual plants, for example, moisture provides a partially reliable cue for future success, and diversification bet hedging may concurrently evolve around norms of reaction, increasing the variance in germination events among progeny of individuals within (Donohue et al., 2005; Simons, 2014) and among seasons (Clauss & Venable, 2000; Cohen, 1966; Evans, Ferriere, Kane, & Venable, 2007; Gremer et al., 2016; Philippi, 1993), thus reducing risk of low or zero parental fitness.

**Figure 1:**
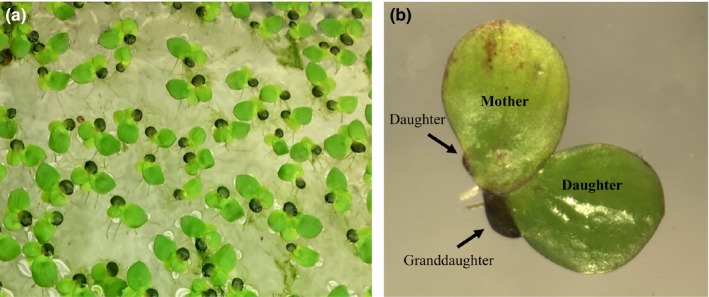
The floating aquatic plant Greater Duckweed, *Spirodela polyrhiza* (a) and a late birth‐order mother producing a regular frond as her first daughter (from the right), and a dense overwintering “turion” frond (from the left), as her second daughter (b). The regular daughter frond is producing a turion granddaughter. Under the same conditions, an early birth‐order mother of the same genotype would produce only regular fronds

*Spirodela polyrhiza* (Araceae or Lemnaceae; taxonomic opinion differs), or Greater Duckweed, is a widely distributed floating perennial plant found in a diverse range of aquatic environments (Figure 1a) (Jacobs, 1947; Ladolt & Kandeler, 1987; Landolt, 1986). Flowering has rarely been observed (Krajncic & Devidé, 1979; Krajncic, Slekovec‐Golob, & Nemec, 1995), and genetic evidence (Tang, Zhang, Cui, & Ma, 2014) also suggests that reproduction occurs almost exclusively through sequential asexual budding of “daughter” fronds from two meristematic pouches (Figure 1b).

*Spirodela polyrhiza* provides an appropriate model system because it relies on seed‐like but vegetative propagules called turions to overwinter (Jacobs, 1947; Ladolt & Kandeler, 1987; Smart & Trewavas, 1983). Turions are produced from the meristematic pouches in place of normal fronds (Jacobs, 1947; Ladolt & Kandeler, 1987; Smart & Trewavas, 1983), are starch‐rich, lack aerenchyma, and are thus dense, sinking to the sediment surface upon production (Jacobs, 1947; Landolt, 1986). Variable phenology of turion formation has been observed in the field (Compton, 2013), and this phenology is expected to be influenced by plasticity to cues, indicative of deteriorating environmental conditions such as low temperatures or deficiency in nutrients; especially phosphate (Appenroth & Nickel, 2010), nitrate and sulfate (Malek & Cossins, 1983) in this photoperiod neutral species (Krajncic et al., 1995).

However, aquatic habitats are also characterized by stochasticity, such as in terminal freeze‐up dates. A physical state change from liquid to solid medium imposes a hard and unpredictable termination of the season. Early turion formation is maladaptive under expected conditions because of lost reproductive potential, whereas late turion formation risks lineage extinction under early onset of lethal conditions. The phenology of turion formation that maximizes fitness is thus unpredictable, and the evolution of diversification bet hedging is expected. The problem for *S. polyrhiza* exemplifies the focus of the recent literature; Namely, how diverse phenotypes might be generated among genetically identical offspring (for a review of developmental mechanisms, see Abley, Locke, & Leyser, 2016).

Parental fronds in the population under study release ten to fifteen offspring (“daughters”) sequentially through budding from two meristematic pouches, although as many as twenty have been observed in other studies (Hillman & Culley, 1978). As in other duckweed species, sequential daughters differ phenotypically, including in diminishing size (Barks & Laird, 2015, 2016; Landolt, 1986; Smart & Trewavas, 1983). This raises the possibility that—in addition to plastic variation in response to environmental cues—diversification in turion phenology may be produced through birth order. Variation in the timing of turion production has been observed across seasons in nature (Appenroth & Nickel, 2010), and has been observed to vary over a range of a few months within single ponds (Compton, 2013) where each frond experiences similar conditions, suggesting that variation in turion production is generated, in addition to extrinsic conditions, by other factors. Variance may be produced by a range of developmental mechanisms (reviewed in Abley et al., 2016), by nonadaptive microplasticity (Bradshaw, 1965; Simons & Johnston, 1997, 2006), or by parental environmental, age, or architectural effects on offspring traits (Castellanos, Medrano, & Herrera, 2008; Galloway, 2001; Wolfe, 1992).

Here, we hypothesize that turion phenology is influenced jointly by plasticity and diversification; specifically, that it responds consistently through plastic response to temperature, and that potential diversification in single genotypes is generated through birth order. Although a direct test of bet hedging is beyond the scope of this study, we aim to provide insight into the evolution of bet hedging by testing hypotheses on how variation is generated among genetically homogeneous individuals (Abley et al., 2016) and on the joint expression of plasticity and potential diversification bet hedging in an emerging model organism (Wang et al., 2014).

## 2 MATERIALS AND METHODS

**Figure 2:**
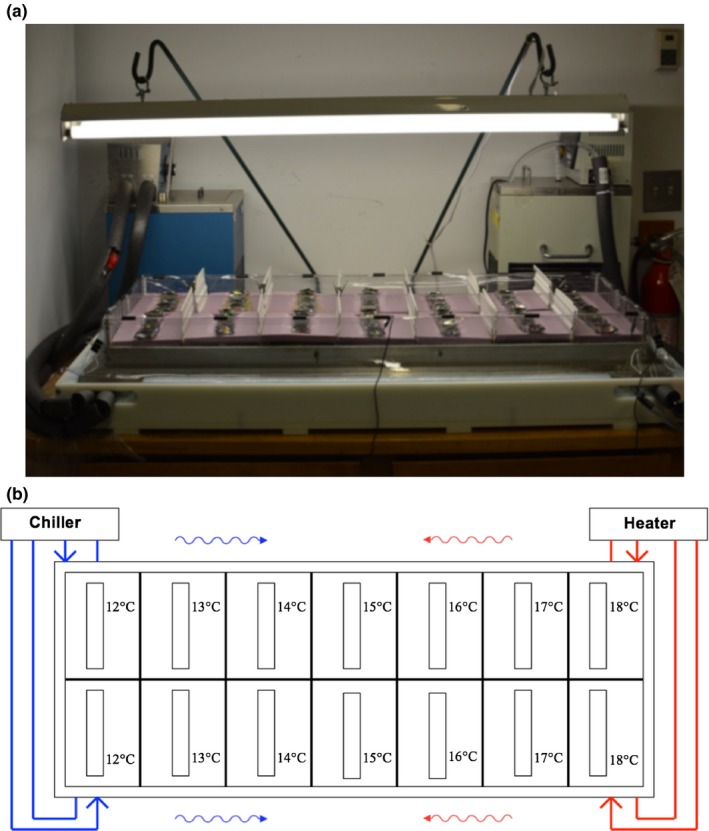
Thermogradient incubator (a) and a schematic of its operation (b). The circulating cold and hot water at opposite ends regulate temperature within a solid aluminum base, and does not enter the upper aquatic chamber containing liquid growth medium. Fronds of *Spirodela polyrhiza* were placed in strainers to separate birth orders at each of the seven temperature positions within the upper aquatic chamber

A thermogradient incubator, generally used to investigate seed germination in response to temperature, (Thompson & Whatley, 1984), is used here to simultaneously assess plasticity to a range of temperatures, and the potential diversifying effects of parental birth order on the phenology of turion formation. The main component of the thermogradient incubator is a solid aluminum block (126 × 46 × 11 cm). A temperature gradient is established in the block through the use of a water chiller that sends cold water in counter‐current through two closely spaced holes drilled through one end of the block, while heated water flows in counter‐current from a water heater through two holes drilled through the opposite end (Figure 2). An aquatic chamber containing Appenroth's liquid growth medium (Appenroth, Teller, & Horn, 1996) is installed on the surface of the aluminum block, and is split by a waterproof barrier into two replicate lanes, allowing for simultaneous runs of a study. Two fluorescent lamps (Symban F32T8/841/ECO) above the thermogradient provided a 14/10 hours photoperiod with a light intensity of 42 μmol m^−2^ s^−1^. White perforated barriers were placed in the aquatic chamber between each of seven temperature positions to reduce eddy flow and stabilize the temperature gradient. Each temperature position, from 12 to 18°C in 1°C increments, was monitored daily using temperature probes. This range was chosen because, under homogenous nutrient conditions in a seed germinator (Biochambers model SG‐30), *S. polyrhiza* was found to produce only vegetative fronds at water temperatures above 18°C and mainly turions at temperatures less than 10°C. The goal was thus to determine plasticity to temperature for different birth‐order mothers over a range that induces the production of both fronds and turions.

Prior to initiating the study, the thermogradient was allowed to stabilize for one week with eight liters of Appenroth's nutrient medium in each replicate lane. The thermogradient was covered to control evaporation of the nutrient medium. Fronds were contained within 2.5 cm diameter strainers supported at the water surface by cutout Styrofoam floats.

Turions collected from the Queen's University Biological Station were reactivated for the thermogradient study by placing them in a 1200 ml crystallization dish with 1 liter of Appenroth's growth medium (Appenroth et al., 1996) in a seed germinator (Biochambers SG‐30). Because the focus of this study is on differences generated within rather than among genotypes, because propagation occurs through budding in the field (Tang et al., 2014), and because logistical constraints preclude following multiple (putative) genotypes within the thermogradient, a single new turion was used to propagate the source pool for *S. polyrhiza* fronds. After reactivation, 84 individual fronds produced from this turion were separated and distributed among seven 12‐well plates. Preparation of fronds for the starting point of the study then required elimination of environmental effects and standardization of effects of birth order by producing first daughters of first daughters (etc.) for at least five generations. Following this standardization, budding of fronds was monitored within the 12‐well plates. Each complete birth‐order sequence (1, 3, 5, 7, 9) was produced by a unique mother. All five required birth orders were separated and transferred to each of two replicate, color‐coded strainers at each of seven temperatures within the thermogradient resulting in a total of 70 strainers (35 per lane) and a sample size of four fronds per strainer giving 280 fronds per thermogradient run, or a total of 560 fronds. Odd birth orders were used because fronds are produced from two pouches almost simultaneously (Jacobs, 1947; Smart & Trewavas, 1983), and paired daughters may behave similarly. Extra samples were maintained in the 12‐well plates in case of frond death.

After placing birth order 1 into the thermogradient, birth order 3 was introduced 5 days later, followed by birth order 5 after 7 days, birth order 7 after 8 days, and birth order 9 after 10 days (30 days after birth order 1). The thermogradient remained stable throughout the study, and the growth medium was replaced at the introduction of each new birth order and once per week thereafter. However, to ensure that temporal factors did not confound birth‐order effects, two approaches were taken. First, an “extension run” was performed immediately following the first run without altering the conditions, but using only birth‐order 1 mothers so that their behavior could be compared to the original birth‐order 1 mothers from the beginning of the run. In addition, a second complete thermogradient run was performed, but as a “backward run” in which birth orders were introduced into the thermogradient in reverse sequence (9, 7, 5, 3, 1). This reverse birth order had to be produced while manipulating the timing of placement into the thermogradient to be similar to that of the forward run. To prepare birth‐order 9 mothers to initiate the backward run, birth orders 1 through 8 were discarded as they were produced in their 12‐well plates. New plates were setup to prepare birth‐order 7 fronds, timed for their correct sequential introduction into the thermogradient, by discarding birth orders 1 through 6. All birth orders were similarly prepared, so that each birth order was transferred to the thermogradient with similar, but reversed, timing compared to the forward run. Thus, unlike in the forward run, daughters were necessarily derived from different mothers, although from mothers of the same clone and with the same birth‐order history. In this run, the medium was replenished once a week and the placement of a new birth order inside the incubator was scheduled so that it would fall on the same day as the medium change. Birth‐order 1 fronds were thus placed into the thermogradient 28 days after birth‐order 9 fronds were first introduced.

Turion production for the different birth‐order mothers was measured as birth order of first turion produced, as days to first turion, and as turion frequency. Once a mother frond produced a daughter, whether a frond or turion, it was gently separated from the mother only upon maturity, that is, after the daughter initiated its own daughter frond. Length and width of all mother fronds were also measured to assess the effect of size on turion phenology. Each mother and daughter produced was photographed using an Olympus SZX12 microscope with an Infinity 3 Lumenera camera using a 0.5× lens at 10× magnification. The digital photographs were calibrated, and frond length (apex to opposite edge) and width (perpendicular to length at widest point) measurements were taken using NIH ImageJ version 1.49v. Here, we use mother width as our size measure because preliminary analyses show that although length and width are strongly correlated (*r* = .83), partial correlations with birth order indicate that the negative relationship between mother size and birth order is accounted for a highly significant partial correlation with width (*r* = –.49; *p* < .001).

### 2.1 Statistical analysis

The effect of mother birth order, mother frond size, temperature, thermogradient run (forward, backward), and their interactions on first turion birth order was determined using ANCOVA. Separate post hoc multiple regression analyses were conducted for the forward and backward runs to further examine the effect of temperature and mother birth order within each run in the event of interactions with thermogradient run. The extension run was used to compare the effects of mother birth order to the forward run. Mother birth order 1 of the extension run was coded as a separate level in an ANCOVA of the effects of mother birth order (nominal) and temperature (continuous) on first turion birth order that included all data from the main thermogradient study and the extension run. A post hoc Tukey's test was then used to compare behavior across birth orders.

Days to first turion and turion frequency (number of turions divided by the total number of daughters, arcsin‐square root transformed prior to analysis) are alternate measures of turion phenology for individual mothers. For completeness, these were similarly assessed by conducting multiple regression analyses of the effect of temperature, mother birth order, and their interactions for the forward and backward runs. The phenology of turion production (first turion birth order and days to first turion) would be seriously biased toward early turion production if turion data for mothers producing no turions during their lifetime were simply missing from the dataset. To accommodate standard analyses consistently across the study, a conservative (i.e., potentially reduces effect size) transformation approach was taken to eliminate the “invisible fraction” bias (Weis, 2017) caused by ignoring only mothers refractory to producing turions: A hypothetical late turion—as a daughter following the final true daughter—was added to the dataset for mother fronds producing no turions in analyses of turion birth order and time to turion production. However, zero turion production does not present a bias problem for turion frequency.

Finally, logistic regression was conducted to predict the effect of temperature, mother birth order, and daughter birth order on daughter state being a frond or turion. All analyses were performed using SAS JMP v.13.

## 3 RESULTS

**Figure 3:**
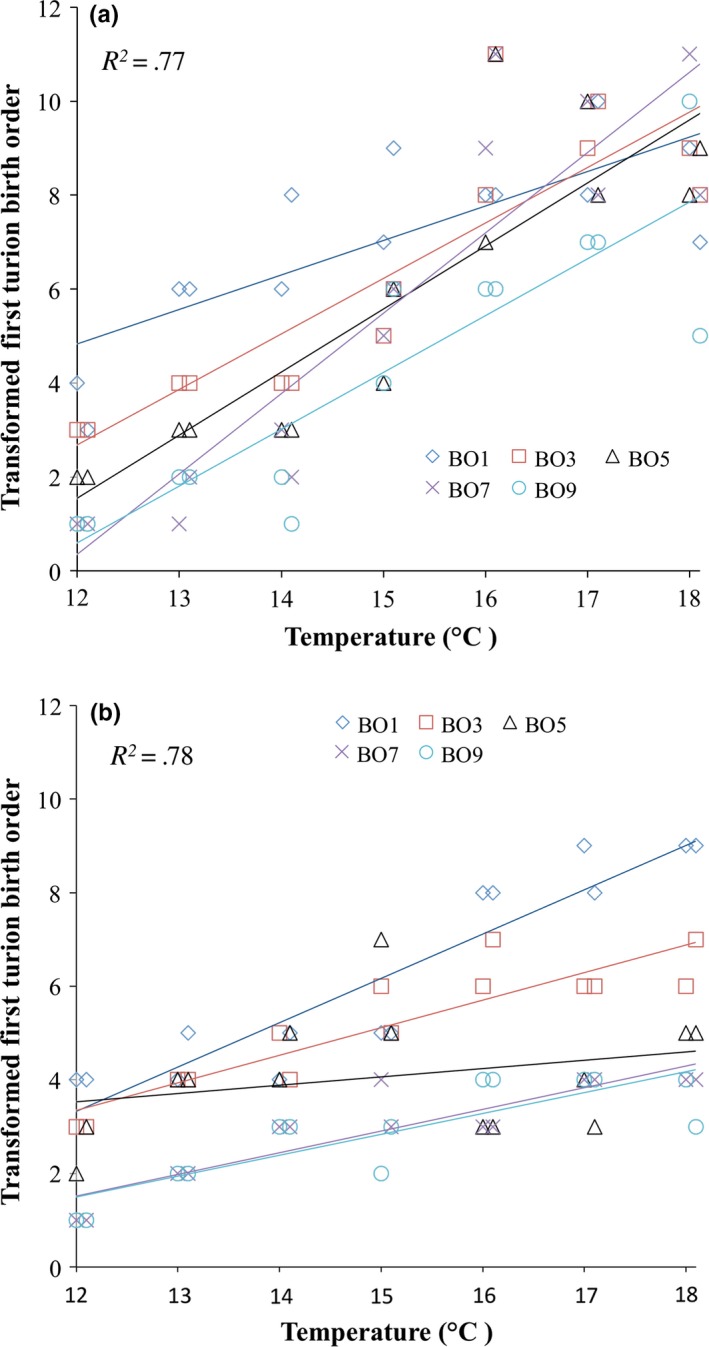
The effects of mother birth order and temperature on transformed (addition of hypothetical turion to refractory mothers; see Methods) first turion birth order from the forward (a) and backward (b) thermogradient runs. The thermogradient incubator is split into two replicates, reflected by the two points for each birth order at each temperature. For display purposes, coincident data points are slightly offset within temperatures. Multiple regression *R* ^2^ values are for the two‐factor models including interactions

The ANCOVA to assess the effects of temperature, mother birth order, size, and thermogradient run on first turion birth order found highly significant effects of temperature and mother birth order, and a marginally significant effect of frond size (Table 1). However, several interactions involving thermogradient run were significant, and because interaction effects confuse the interpretation of main effects (Gotelli & Ellison, 2013), post hoc analyses were performed to examine the effects of mother birth order and temperature in the two individual runs in more detail. The main effects remain highly significant in the post hoc analysis (mother birth order and temperature, both runs: *p *<* *.0001), and their interaction becomes significant (Forward: *p *=* *.017; backward: *p *=* *.0048); it is clear (Figure 3) that higher birth‐order mothers produce turions sooner than do mothers of low birth order in both thermogradient runs. For example, in both runs, birth order 9 consistently produced a first turion early across all temperatures, followed by birth order 7, 5 and 3, while birth order 1 mothers made a first turion the latest on average (Figure 3). These results remain qualitatively unaltered if frond size and all interactions are included as follows: A strong effect of birth order on turion phenology exists beyond that explained by size, with size also showing a significant effect but only in the backward run (*p *=* *.01).

**Table 1:** ANCOVA for the effects of temperature, mother birth order, size (frond width), and thermogradient run (TGR) on first turion birth order

Source	*df*	SS	*F*‐ratio	*p*
TGR	1	51.10	37.25	<.0001
Temperature	1	167.7	122.3	<.0001
Mother birth order	1	15.57	11.35	.001
Size	1	5.533	4.033	.047
TGR*Temperature	1	73.35	53.47	<.0001
TGR*Mother birth order	1	1.058	0.7708	.382
TGR*Size	1	1.074	0.7827	.378
Temperature*Mother birth order	1	0.5125	0.3736	.542
Temperature*Size	1	0.9668	0.7047	.403
Mother birth order*Size	1	0.2025	0.1476	.702
TGR*Temperature*Mother birth order	1	8.178	5.961	.016
TGR*Temperature*Size	1	2.859	2.084	.151
TGR*Mother birth order*Size	1	1.789	1.304	.256
Temperature*Mother birth order*Size	1	0.1798	0.1310	.718
TGR*Temperature*Mother birth order*Size	1	6.600	4.811	.030

Analysis of covariance—with temperature as the covariate—comparing the “extension” run (using only birth order 1 mothers) and the forward run finds that significant differences in first turion birth orders among mother birth orders (DF = 5; *F* = 13.52; *p* < .001) occur between mother birth order 1 of the extension run and all mother birth orders (3, 5, 7 and 9) except birth order 1 of the main thermogradient study in a post hoc Tukey's test.

**Figure 4:**
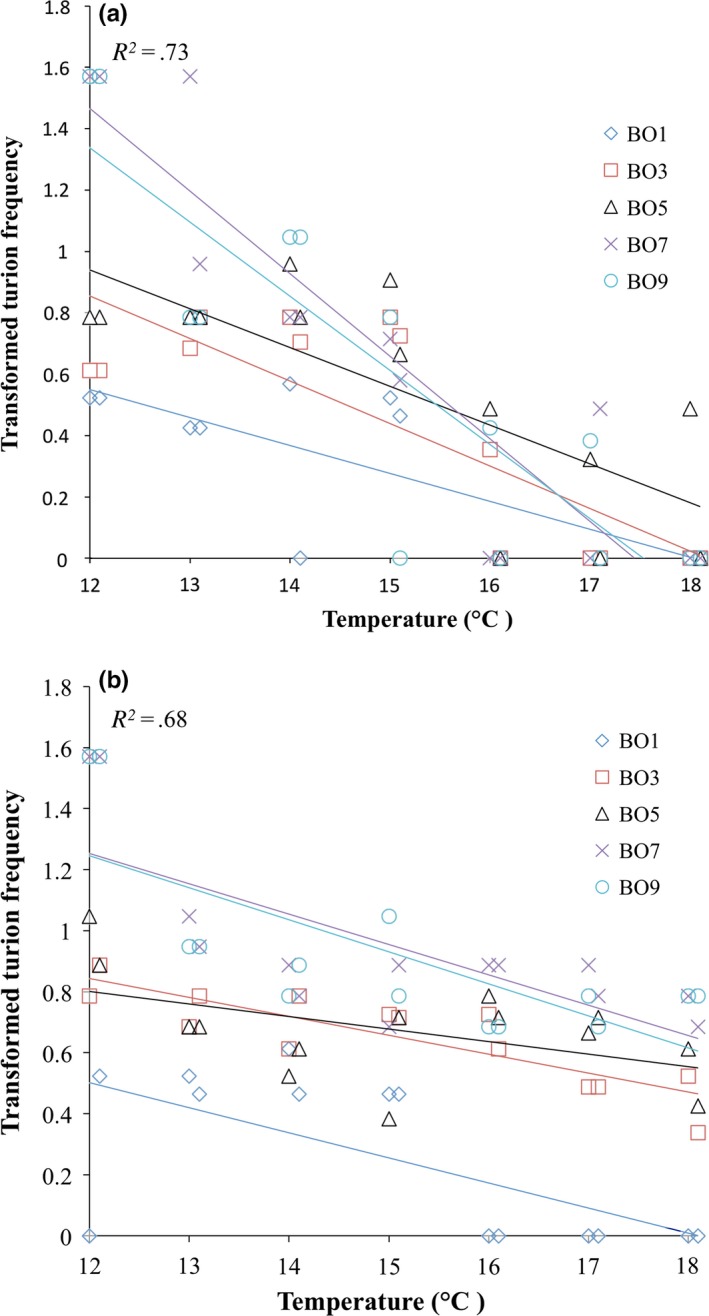
The effects of mother birth order and temperature on arcsin‐square root transformed turion frequency from the forward (a) and backward (b) thermogradient run. The thermogradient incubator is split into two replicate lanes, reflected by the two points for each birth order at each temperature. For display purposes, coincident data points are slightly offset within temperatures. Multiple regression *R* ^2^ values are for the two‐factor models including interactions

Alternate measures of turion phenology and production are the time taken to produce a first turion, and the frequency of turion formation. Experiment‐wise, individual mother fronds produced between zero and five turions (mean = 1.7; median = 2), with a substantial proportion producing two (29%) or three (24%) turions, with only 5% producing four or five. Multiple regression analyses to assess the effects of temperature, mother birth order, and their interactions on days to turion production (Table 2) and transformed turion frequency (Figure 4) are qualitatively consistent with the above results for first turion birth order, finding significant main effects of mother birth order and temperature with the exception that no temperature effect on days to turion production is found for the backward TGR (Table 2). In addition to the main effects of temperature (both runs: *p *<* *.001) and birth order (both runs: *p *<* *.001) on turion frequency, Figure 4 clearly illustrates an interaction effect in the forward (*p *<* *.001; Figure 4a), but not backward (*p *=* *.277; Figure 4b) TGR. Running the full model including mother frond size and thermogradient run (as in Table 1) again yields consistent results for both days to turion formation and transformed turion frequency, with significant effects of temperature and mother birth order; however, the effect of frond size—which was marginally significant for first turion birth order—is nonsignificant for both alternative response variables (days to first turion: *p *=* *.130; turion frequency: *p *=* *.913).

**Table 2:** Multiple regression of the effects of temperature and mother birth order on days to first turion shown separately for the forward thermogradient run and backward thermogradient run

Thermogradient run	Effect	*df*	Sum of squares	*F*‐Ratio	Probability
Forward	Temperature	1	5228.93	62.05	<.0001
	Mother birth order	1	352.03	4.18	.0450
	Temperature*Mother birth order	1	497.83	5.91	.0178
Backward	Temperature	1	55.80	0.99	.3226
	Mother birth order	1	4537.21	80.74	<.0001
	Temperature*Mother birth order	1	88.80	1.58	.2132

**Figure 5:**
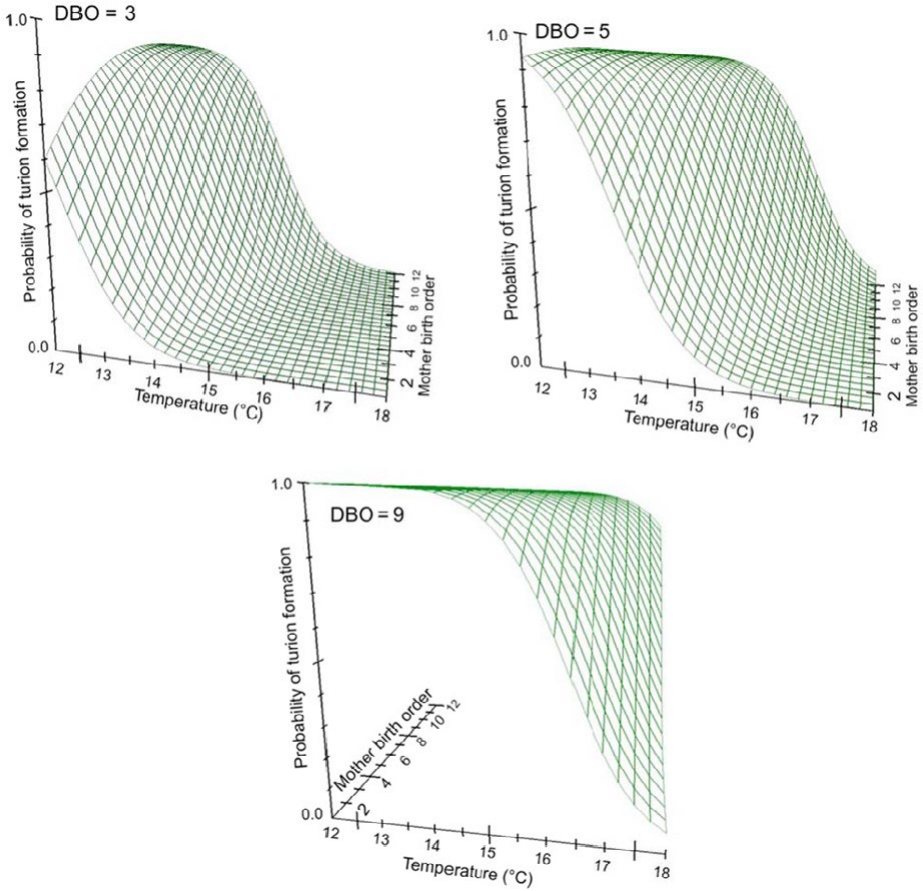
The probability of turion formation across temperatures and mother birth orders converted from the log odds function of the logistic regression. Turion formation also depends on daughter birth order (a fourth dimension); thus, for the purposes of illustrating on 3D graphs, this relationship is shown for three daughter birth orders (DBO = 3, 5, 9)

As a final analysis of turion phenology, a logistic regression was used to model the equation predicting the type of daughter (vegetative frond or turion) produced as a function of mother birth order, daughter birth order, and temperature. All effects are strong predictors of the formation of turions (Table 3). Based on our data, and over the range of temperatures observed, the log odds of frond:turion formation, or logit function, is ln[p(frond)/p(turion)] = −16.31 + 1.64(°C) – 0.41(mother birth order) – 1.12(daughter birth order). Taking the inverse of exp(logit function) and converting from odds ratio gives the probability of turion formation as a function of temperature and birth order (Figure 5).

**Table 3:** Logistic regression of the effects of temperature, mother birth order, and daughter birth order on predicting turion formation

Effect	Parameter estimate	Wald chi‐squared test	*df*	*p*
Intercept	–16.31	68.33	–	<.0001
Temperature	1.64	84.69	1	<.0001
Mother birth order	–0.41	35.77	1	<.0001
Daughter birth order	–1.12	84.90	1	<.0001

## 4 DISCUSSION

Phenotypic plasticity and bet hedging are two evolutionary modes of response to varying environmental conditions, where adaptive plasticity may evolve in response to cues (Bradshaw, 1965; Stearns, 1989), and bet hedging evolves to the extent that cues are unreliable indicators of future success, or the evolution of plasticity is constrained (Seger & Brockmann, 1987; Simons, 2011). Although plasticity evolves through selection on norms of reaction, and thus requires genotype‐environment interaction (Via & Lande, 1985), the mechanisms generating individual‐level phenotypic variance as diversification bet hedging remain obscure.

Here, we use *Spirodela polyrhiza* for an enquiry into mechanisms generating individual‐level diversification. It is a suitable system because variation among its clonally produced offspring cannot be a product of genetic variation, and because it inhabits variable environments in which the timing of turion formation is expected to have direct fitness consequences. Early turion formation reduces potential fitness through reduced reproduction, but if turion formation is delayed, there is a risk of zero overwinter survival. Field observation confirms plasticity to environmental conditions including temperature (Hillman, 1961; Jacobs, 1947), but variable turion production observed in the field (Compton, 2013) does not necessarily imply individual‐level diversification and has several potential sources. We ask whether genetically identical individual duckweed fronds produce phenological variation in turion production through birth order, and to what extent plasticity and potential diversification bet hedging are coexpressed.

Our findings suggest that mother birth order, both through its direct effect and its interaction with temperature on first turion birth order, is an important source of potential bet‐hedging diversification. The results are robust to choice of measure of turion phenology as the response variable, in that birth order at which a first turion is produced, days to first turion, and frequency of turions produced by a parent all lead to similar conclusions about effects of mother birth order and temperature. The one exception—the finding of no significant effect of temperature on days to first turion production in the “backward run”—is likely explained by the direct effect of temperature on development time and frond production, although the difference in significance level of the effect of temperature between the two runs lacks an explanation.

Overall, the effect of temperature suggests that mothers may adjust turion phenology according to predictable seasonal variation, producing turions as temperatures diminish. However, plasticity to temperature alone will generate little variation in phenology within a season, because all fronds would switch simultaneously to turion production once a threshold temperature was reached in a pond experiencing cooling conditions. It should be noted that water temperature and phosphate availability may vary on a fine spatial scale within natural ponds (Schlosser, 1990), and likely generates some plastic variation in turion formation. However, because fronds experience only surface waters, the extent of spatial variation in temperature cues is minor compared to diurnal and seasonal changes occurring from midsummer through freeze up in the late fall.

Because of the unique reproductive pattern of overlapping generations expressed by duckweed, in which daughter fronds begin producing their own sequence of daughters often while still attached to the parent frond (Lemon & Posluszny, 2000), every birth order may be present at all times throughout the season. Parental birth order will cause variation in turion phenology because mothers of all birth orders of the same genotype are present as a pond changes temperatures through the season, with early birth‐order mothers continuing to produce regular fronds even under cool conditions at the same time that late birth‐order mothers produce turions. Thus, our results suggest that plasticity to cues and diversification through birth order are coexpressed to produce observed variation in the timing of turion formation. Furthermore, the observed range in turion formation phenology produced by birth order within temperatures is similar to that expressed as plasticity to temperature, suggesting ecological relevance of both factors. A multigenerational study that includes also grandparental effects would shed light on the longer‐term consequences of birth order on variation in dormancy behavior. In addition, molecular genetic analysis could ask whether more than one genotype exists at a location and whether populations harbor genetic variation in both the timing of turion formation and its plasticity.

The intention here was to identify potential sources of individual‐level variation in phenology as potential diversification for a range of environments over which plasticity is also expressed. The results highlight several areas in need of further investigation. First, variation in turion phenology provides only a mechanism for diversification but cannot be interpreted as diversified bet hedging unless it is shown to increase long‐term fitness (Philippi & Seger, 1989). Tests of bet‐hedging theory are notoriously difficult to perform because fitness effects must be documented over multiple generations (Simons, 2011). In this instance, a test of optimal bet hedging would require an assessment of the fitness consequences of observed variance in phenology of turion formation over multiple seasons in which freeze‐up date of ponds is representative of variation over an evolutionarily relevant timescale. The inclusion of adaptive plasticity in an optimality test would further require assessment of the fitness consequences of genotypic norms of reaction.

Second, norms of reaction in turion formation isolate effects of temperature in this study, but are not meant to imply that no other cues exist. Studies of cues, especially the decrease in phosphate levels as assessed by Appenroth and Nickel (2010), would add to our understanding of the relationship between plasticity and diversification. Although the balance between plasticity and bet hedging cannot be rigorously quantified here, we can conclude that the effects of birth order and temperature play substantive roles in turion formation, indicating the importance of the joint expression of these two modes of response in the field.

Third, although mother birth order is found to generate variation in turion formation phenology, the mechanistic source of this turion variation remains unknown. However, previous studies have reported the effects of mother age on offspring frond quality (Barks & Laird, 2015), which could be a result of maternal anatomical variation in such factors as stipe tissue build up near the meristematic pouches (Lemon & Posluszny, 2000). The existence of phenotypic variation across birth orders may reduce the reliance on alternative proposed variance‐generating mechanisms such as epigenetic patterning (Bonduriansky, Crean, & Day, 2012; Herrera, Medrano, & Bazaga, 2014) and molecular and developmental stochasticity (for review, see Abley et al., 2016) in this particular system, but these mechanisms may act to fine‐tune variance expression (Maxwell & Magwene, 2017) and are certainly worth investigating given the extent of phenological variation observed among genetically identical individuals.

Fourth, this study of the phenology of turion formation addresses mechanisms of coping with the onset of winter, but another component of uncertainty in the *S. polyrhiza* life cycle is the optimal timing of springtime reactivation of turions that have been previously formed. It is, at present, unknown whether turion reactivation in the spring is determined exclusively by temperature and light cues (photoperiod, intensity, quality), by time elapsed since production, by turion birth order, or by other factors, and is the subject of ongoing research.

The duckweed system provides opportunities to further test variance strategies, as well as life history theory more broadly. For example, Barks and Laird (2016) demonstrated that non–turion‐producing *Lemna* spp. exhibit diminishing fitness with birth order; if this holds for *S. polyrhiza*, the switch to turion production in late birth‐order fronds with decreasing residual reproductive value may be a low‐cost solution for variance generation. Interest in the adaptive significance of the expression of individual phenotypic variance (Bull, 1987; Simons & Johnston, 1997) has recently intensified (Abley et al., 2016; New et al., 2014; Rossi, Gandolfi, & Menozzi, 2017; Stelkens, Miller, & Greig, 2016; Xue & Leibler, 2016), and has become topical in fields such as medicine (*e.g.,* Rovira‐Graells et al., 2012) and human psychology (Hertler, 2017). The effects of birth order on life history phenology observed in the present study provide an additional source of individual, or within‐genotype variance expression; however, inferences about the adaptive significance of this phenological variation in the field will require further study.

## AUTHOR CONTRIBUTIONS

Both authors contributed equally to this work. H.S.M. and A.M.S. designed the study, developed the methodology, performed the analysis and wrote the manuscript. H.S.M. performed the experiments.
